# Gut and lung microbiome profiles in pregnant mice

**DOI:** 10.3389/fmicb.2022.946779

**Published:** 2022-12-12

**Authors:** Rosana Wiscovitch-Russo, Aji Mary Taal, Claire Kuelbs, Lauren M. Oldfield, MohanKumar Ramar, Harinder Singh, Alexey V. Fedulov, Norberto Gonzalez-Juarbe

**Affiliations:** ^1^J. Craig Venter Institute, Rockville, MD, United States; ^2^Department of Surgery, Division of Surgical Research, Rhode Island Hospital, The Warren Alpert Medical School of Brown University, Providence, RI, United States

**Keywords:** microbiome, pregnancy, stool, lung, 16S sequence

## Abstract

In recent years, microbiome research has expanded from the gastrointestinal tract to other host sites previously thought to be abacterial such as the lungs. Yet, the effects of pregnancy in the lung and gut microbiome remains unclear. Here we examined the changes in the gut and lung microbiome in mice at 14 days of gestation. Lung tissue and stool samples were collected from pregnant and non-pregnant female BALB/c mice, DNA was isolated, amplified, and bacterial specific V4 16S rRNA gene was sequenced. Using an in-house bioinformatic pipeline we assessed the microbial composition of each organ using stool and lung tissue samples. The stool data showed that Lachnospiraceae and Lactobacillaceae were more abundant in the pregnant mice. Likewise, Lactobacillaceae were dominant in the lungs of pregnant mice. However, Streptococcaceae were dominant in the lungs of non-pregnant mice with a low microbial abundance in the pregnant mice. A permutation test showed that pregnancy significantly contributes to the variance in both the lung and stool microbiome. At the same time, we estimate that 49% of the total detected operational taxonomic units were shared between the stool and lung data. After removing common stool-associated bacteria from the lung dataset, no microbial differential abundance was detected between the pregnant and non-pregnant lung microbial community. Thus, pregnancy contributes to variance to the lung and stool microbiome but not in the unique lung microbiota.

## Introduction

Microbiome research has recently focused on studying the influence of microbial populations from distal host sites in relation to host–microbe interactions and development of disease. To date the gut-brain axis remains the most studied inter-organ interaction, however, recent studies are now focused into the role of the microbiota in the gut-lung axis (Enaud et al., 2020). The lung and gut microbiota can modulate local host immune responses. Alterations in the lung or gut microbiome have been shown to influence the course of respiratory diseases such as acute respiratory infections, chronic obstructive pulmonary disease, asthma, or cystic fibrosis (Madan et al., 2012; Trompette et al., 2014; Nielsen et al., 2016; Sprooten et al., 2018; Trompette et al., 2018). The crosstalk between the lung and gut microbiota can have a local or distant effects that can be driven by live bacterial cells, bacterial derived fragments like lipopolysaccharides (LPS) or metabolites like short-chain fatty acids (SCFAs) (Sze et al., 2014; Trompette et al., 2014; Qian et al., 2018; Wedgwood et al., 2020). These microbial byproducts can translocate from distant mucosal sites (gut or lung) through the host circulatory system providing a positive or negative impact on the host overall health (Trompette et al., 2014; Qian et al., 2018). Essentially, the gut-lung axis is a two-way communication mostly facilitated by microbial and host immune interactions influencing the host health.

Pregnant women are considered a special population since they are at higher risk of developing infectious diseases (Mor and Cardenas, 2010) and have a number of distinct immunologic features, e.g., Th2-predominance (Wang et al., 2020). During pregnancy, hormonal changes produce physical, immunological and metabolic adaptation to support the development and avoid rejection of the fetus (Koren et al., 2012; Elderman et al., 2018; Faas et al., 2019). For instance, placenta derived hormones produce an insulin resistant state to store energy in the maternal adipose tissue (Parrettini et al., 2020). Gains in adiposity during pregnancy generates higher levels of leptin, insulin, and cholesterol (Koren et al., 2012). Significant increase in these metabolites during mid- to late-pregnancy can lead to metabolic disorders such as type 2 diabetes and preeclampsia (Salzer et al., 2015). Overall, these hormonal and immunological adaptations influence the microbiome and therefore the production of secondary metabolites (Koren et al., 2012; Elderman et al., 2018; Faas et al., 2019). Changes during pregnancy may also increase the risk of disease associated to microbial dysbiosis (Koren et al., 2012; Amir et al., 2020; Ishimwe, 2021). Moreover, pregnancy has also been associated with specific susceptibility to bacterial (e.g., urinary tract and gut) and yeast (e.g., candidiasis) infections (Kalinderi et al., 2018; Rac et al., 2019). Although considered less common, respiratory infections can occur during pregnancy such as Influenza virus and most recently SARS-CoV-2, the causative of COVID-19 (Laibl and Sheffield, 2006; Kourtis et al., 2014; Ezechukwu et al., 2021). Given that most of these illnesses are driven by hormonal and immunological changes, more studies are needed to assess the role of pregnancy in microbiome alterations especially in the lung and gut environment where crosstalk of the microbial communities can influence the host health (Enaud et al., 2020). In this study, we characterize and compare the lung and gut microbiomes of pregnant and non-pregnant female BALB/c mice to elucidate the relationship between microbiota from the gut and lung.

## Materials and methods

### Mice and sample collection

Six-to-nine-week-old female BALB/c mice were purchased from Charles River Laboratories (Wilmington, MA). The experiment was conducted in accordance with the Institutional Animal Care and Use Committee approved by the Rhode Island Hospital, IACUC #504718. The mice were housed in a specific pathogen –free (SPF) barrier facility and fed *ad libitum* commercial pelleted mouse food and water. When mice pregnancy is distinguishable at 14 days of gestation (third trimester), stool and lung tissue samples from pregnant and non-pregnant female mice were collected under sterile conditions as previously described (Fedulov et al., 2005, 2008). For the lung tissue samples, the mice were euthanized by intraperitoneal injection of Fatal-Plus, the fur was rinsed with alcohol, the chest wall was washed with saline, and lungs were extracted. Samples were washed in sterile ice-cold saline and immediately stored at −80°C after sample collection.

### DNA extraction

Procedure was carried out in a disinfected Class II Biosafety cabinet treated with Eliminase (Decon Labs Inc., PA,) and 70% ethanol afterward exposed to UV light for 20–30 min. DNA from the mice stool and lung tissue samples was extracted using QIAGEN DNeasy PowerSoil Pro Kit (Cat# 47016, QIAGEN, Hilden, Germany). However, alterations were made to steps 1–4 of the PowerSoil protocol to extract DNA from the lung samples lysed in Sigma Aldrich Stabilyser Reagent (Cat# PNS1010, Sigma Aldrich, St. Louis, CA). The Stabilyser reagent is a lysis buffer with additional components to preserve nucleic acid and proteins, ideal for creating tissue lysate stocks for downstream multiomics applications. Since the reagent is a thick solution, tissue was successfully lysed by exchanging the provided PowerBead Pro tubes with ceramic 2.8 mm PowerBead Tubes (Cat# 13114-50, QIAGEN, Hilden, Germany). To control potential contamination, the net weight of the lung tissue samples was determined. Depending on the sample weight, 5 μl of Stabilyser reagent was added per mg of sample, creating 4-fold concentrated tissue lysate less than the recommended amount by the protocol. Samples were mechanically lysed using QIAGEN PowerLyzer at 3000 rpm for 30 s. After lysing, 200 μl of the tissue lysate was aliquoted in the provided PowerSoil microcentrifuge tube and the remaining lysate was stored in −80°C. The 200 μl of tissue lysate was mixed with 200 uL of CD1 buffer. Then PowerSoil protocol was carried out from steps 5–17 without further modifications. DNA was eluted in 50 μl of C6 buffer (10 mM Tris). DNA concentration and quality was measured using through Qubit 1X dsDNA Assay (Cat# Q33231, Thermofisher Scientific, Waltham, MA) and Nanodrop ND-1000 Spectrophotometer at 260/280 nm.

### V4 16S amplification and sequencing

Genomic DNA (gDNA) extracted from mice stool and lung tissue samples was used to amplify the hypervariable V4 region of the 16S rRNA gene. The primers used were from variable region 515-533F forward (GTGCCAGCMGCCGCGGTAA) and 806-787R reverse (GGACTACHVGGGTWTCTAAT), including the Illumina adaptor sequence with the 8 unique base pairs (Freire et al., 2020). Approximately, a total of 100 ng of gDNA was used for PCR reaction with ThermoFisher Platinum Taq DNA Polymerase (Cat# 10966–026, Life Technologies, Carlsbad, CA); 94°C for 5 min, 94°C for 30 s, 55°C for 30 s, 72°C for 30 s for 35 cycles, 72°C for 7 min on a SimpliAmp Thermal Cycler (Cat# A24811, Thermofisher Scientific, Waltham, MA). Libraries were purified using QIAquick PCR purification kit (Cat# 28106, QIAGEN, Hilden, Germany) then quantified using Qubit 1X dsDNA HS Assay. Afterwards libraries were manually normalized and pooled. The fragment size was inspected using High Sensitivity DNA Kit (Cat# 5067–4,626, Agilent, Santa Clara, CA). Additional cleanup was required with the SPRIselect beads at a 1X ratio (Cat# B23318, Beckman Coulter, Pasadena, CA) to remove a small concentration of primer dimers. Removing the primer dimers from the library pool will reduce selectivity of the Illumina System sequencing shorter reads (sequencing bias) ensuring sequencing of the desired V4 16S PCR product (~400 bp). Finally, the library pool was loaded onto a V3 chemistry kit (2×300 bp) and sequenced on Illumina MiSeq system as instructed by the manufacturer (Cat# MS-102-3,003, Illumina Inc., La Jolla, USA).

### Bioinformatic pipeline

Operational taxonomical units (OTUs) were assigned using an in-house bioinformatic pipeline supported by mothur (Schloss et al., 2009) and Uparse (Edgar, 2013). The SILVA 16S rRNA database (version 123) was used to assign OTUs at 97% sequence similarity (Quast et al., 2013). Quality control and filtering of the raw sequence data was performed by KneadData, removing poor quality reads, primer trimming and eliminating host contaminating sequences from mitochondrial 16S rRNA (Pereira-Marques et al., 2019). R statistical platform was used along with phyloseq (McMurdie and Holmes, 2012) and ggplot 2 (McMurdie and Holmes, 2012; Singh et al., 2020) package to generate relative abundance and diversity measure plots. Phylosmith (Smith, 2019) package was used for screening and sub-setting unique OTUs in the data. In addition, Tax4Fun (Asshauer et al., 2015) package was used for functional prediction of the lung and stool microbial community.

#### Data sharing

The raw sequencing data has been deposited to NCBI BioProject under the accession number PRJNA837989.

## Results

To examine the differences in lung and gut microbiome of pregnant and non-pregnant female mice at 14 days of gestation we used 16S rRNA sequencing. With a total of 55 samples (*n* = 29 lungs and *n* = 26 stools) that passed sequence quality control and filtering of Kneaddata software, for each sample type we had a total of 29 pregnant (*n* = 15 lungs and *n* = 14 stools) and 26 non-pregnant (*n* = 14 lungs and *n* = 12 stools) samples. Overall, we observed a total of 926 operational taxonomic units (OTUs). 561 OTUs were identified in the lung tissue samples and 792 OTUs in the stool samples. Because lung tissue samples are of low microbial biomass, the samples are easily susceptible to contamination, negative controls were included in the DNA batch extractions for both the stool and lung samples (Dickson et al., 2018; Wiscovitch-Russo et al., 2022). For the DNA extraction controls, a total of 88 OTUs were identified, the majority were assigned to taxa related to reagent contamination from the DNA extraction kit such as *Sphingomonas*, *Pseudomonas*, *Ralstonia* and *Methylobacterium* sp. (Supplementary Figure S1; Salter et al., 2014; Glassing et al., 2016). The taxon identified in the extraction negative controls were removed, reducing the total number of assigned OTUs to 911 (*n* = 546 lung and *n* = 790 stool data).

### Comparing the stool and lung microbiome of pregnant and non-pregnant mice

**Figure 1:**
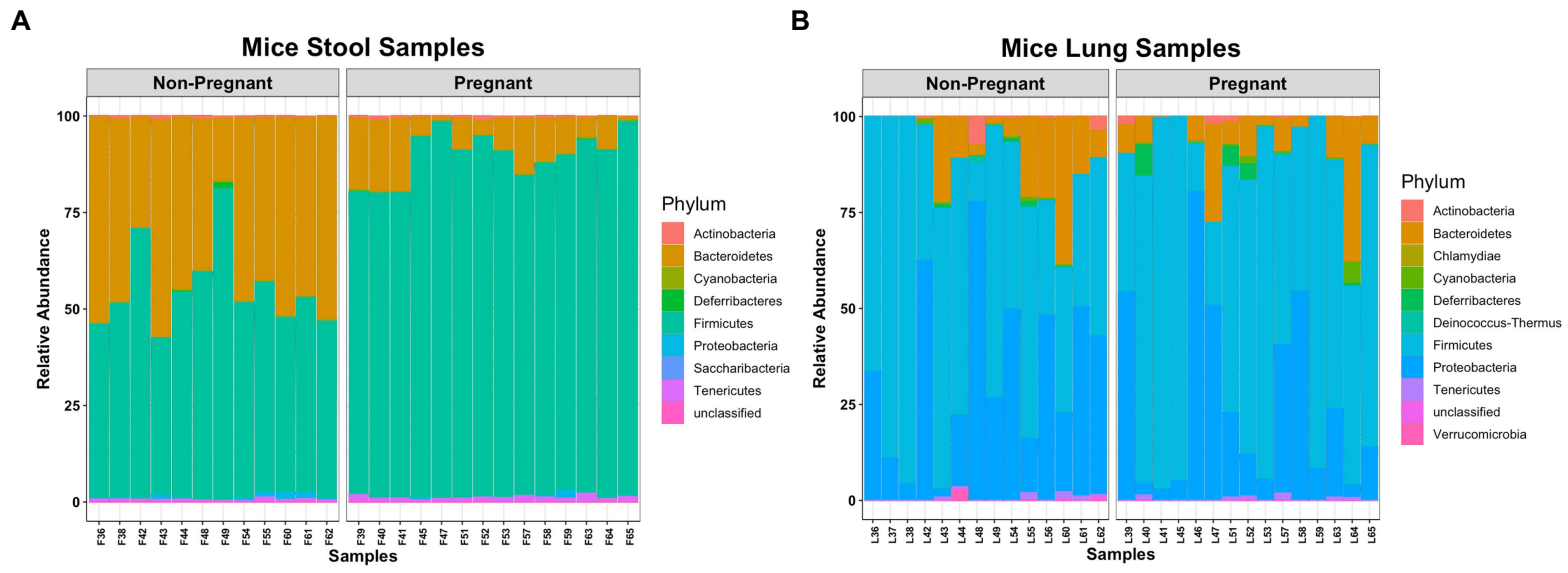
Comparing microbial composition at Phylum level of pregnant and non-pregnant mice. **(A)** Bacteroidetes and Firmicutes were more abundant in stool samples. **(B)** Firmicutes, Proteobacteria and Bacteroidetes were more abundant in lung samples.

The microbial composition between lung and stool samples were distinct. Bacteroidetes and Firmicutes compose most of the fecal microbial phyla (Figure 1A), while in the lungs the major phyla are Firmicutes, Proteobacteria and Bacteroidetes (Figure 1B). In stool, pregnancy led to an increase of Firmicutes over Bacteroidetes. In the lung, pregnancy did not generate differences at phylum level. Interestingly, a low abundance of obligated anaerobic bacterium, Verrucomicrobia (He et al., 2017), was identified among the lung samples.

**Figure 2:**
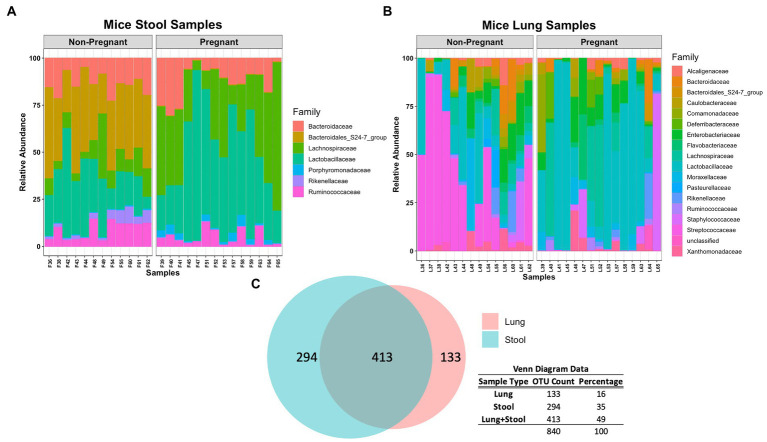
Comparing microbial composition at Family level of pregnant and non-pregnant stool and lung data. **(A)** Lachnospiraceae and Lactobacillaceae were more abundant in pregnant mice whereas Bacteroidales, Rikenellaceae and Ruminococcaceae were more abundant in non-pregnant mice stool samples. Similar to pregnant mice stool samples, Lactobacillaceae are more abundant in pregnant mice lung samples. **(B)** Lactobacillaceae was more abundant in pregnant mice whereas Streptococcaceae was more abundant in non-pregnant mice lung samples. **(C)** Venn diagram demonstrates that lung and stool data share about 413 OTUs in common, while 133 OTUs are unique to the lung data and 294 OTUs are unique to the stool data.

At lower taxonomical classification, the microbial composition between the lung and stool data became more apparent. A closer inspection of the stool microbial composition at Family level shows that Lachnospiraceae and Lactobacillaceae were more abundant in pregnant mice whereas Bacteroidales, Rikenellaceae and Ruminococcaceae were more abundant in non-pregnant mice (Figure 2A). A significant increase of Lachnospiraceae (groups NK4A136 value of *p* <0.0001 and UCG-001 value of *p* <0.01), Ruminococcaceae (group UCG-014 value of *p* <0.05) and *Parabacteroides* sp. (value of *p* <0.001) was observed in the stool of pregnant compared to non-pregnant mice (Supplementary Figure S2). Additionally, *Lactobacillus* sp. was partially increased in the pregnant group (Supplementary Figure S2). The non-pregnant stool data showed significant (value of *p* < 0.001) increase in both *Alistipes* and *Ruminococcus* sp. (Supplementary Figure S2). Noticeably, non-pregnant mice had a higher abundance of unclassified and uncultured bacteria (Supplementary Figure S2). The higher abundance of unclassified and uncultured bacteria in the non-pregnant stool samples reflects further differences in the microbial community driven by pregnancy. Overall, differences in taxonomical classification observed among the pregnant and non-pregnant mice, suggests pregnancy is a major driver for global microbial remodeling in the gut.

The lung data showed a major shift in abundance from Streptococcaceae in non-pregnant mice to Lactobacillaceae in the pregnant group (Figure 2B). More specifically, a significant increase of *Lactobacillus* sp. (value of *p* < 0.05) was observed in the pregnant mice whereas *Streptococcus* sp. (value of *p* < 0.001) was significantly increased in the non-pregnant mice (Supplementary Figure S3). Of interest, Lachnospiraceae and Lactobacillaceae was abundant in both pregnant lung and stool data (Figures 2A,B). In fact, comparing the microbial composition of the stool and lung data revealed these sample types share 413 OTUs in common (Figure 2C). The lung microbiome in mice shares most of its composition with common stool-associated taxa. Thus, it is predicted that a unique lung microbial community that differs from gut-associated bacteria can be found in the observed 133 OTUs.

**Figure 3:**
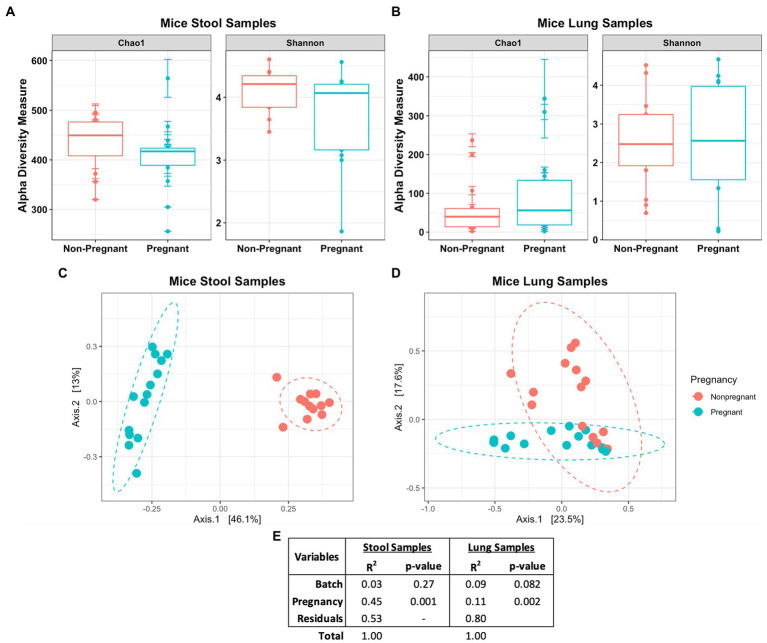
Diversity measures of pregnant and non-pregnant mice stool and lung samples. No significant difference was observed in the Chao1 and Shannon diversity index between the pregnant and non-pregnant groups in either the **(A)** stool or **(B)** lung samples. The unweighted Bray-Curtis PCoA shows the differences between the pregnant and non-pregnant groups in the **(C)** stool and **(D)** lung samples. **(E)** Statistical summary of the PERMANOVA test shows the variance and significance of the pregnancy and DNA batch extraction variables.

Diversity analysis revealed distinctive features in the microbial community between the pregnant and non-pregnant samples. The alpha diversity index measures the observed difference in species richness (number of different species in a community). In the Chao1 and Shannon diversity index no significant differences was observed between the pregnant and non-pregnant groups in either sample types (Figures 3A,B). Chao1diversity index showed that pregnant mice stool had a trend towards lower alpha diversity compared to the non-pregnant mice (Figure 3A). While in the lung samples, pregnancy led to a minor increase in species richness (Figure 3B), the observed differences could correspond to the large abundance of *Lactobacillus* sp. in the pregnant stool samples (Supplementary Figure S2) and *Streptococcus* sp. in the non-pregnant mice lung samples (Supplementary Figure S3). The beta diversity index measures species diversity between two communities, in this case, pregnant and non-pregnant mice. Tight clustering was observed for the pregnancy variable in mice stool samples (Figure 3C), whereas slight clustering was observed in the lung samples (Figure 3D) *via* unweighted Bray-Curtis principal component analysis (PCoA). The Permutational Multivariate Analysis of Variance (PERMANOVA) test showed that pregnancy strongly influenced the gut microbiome (*R*^2^ = 45% and value of *p* = 0.001), and only had a small influence on the lung microbiome (*R*^2^ = 11% and value of *p* 0.002; Figure 3E). Overall, pregnancy-driven changes in microbial communities were observed in both the lung and the stool data.

### Evaluating the suggested lung community unique OTUs

**Figure 4:**
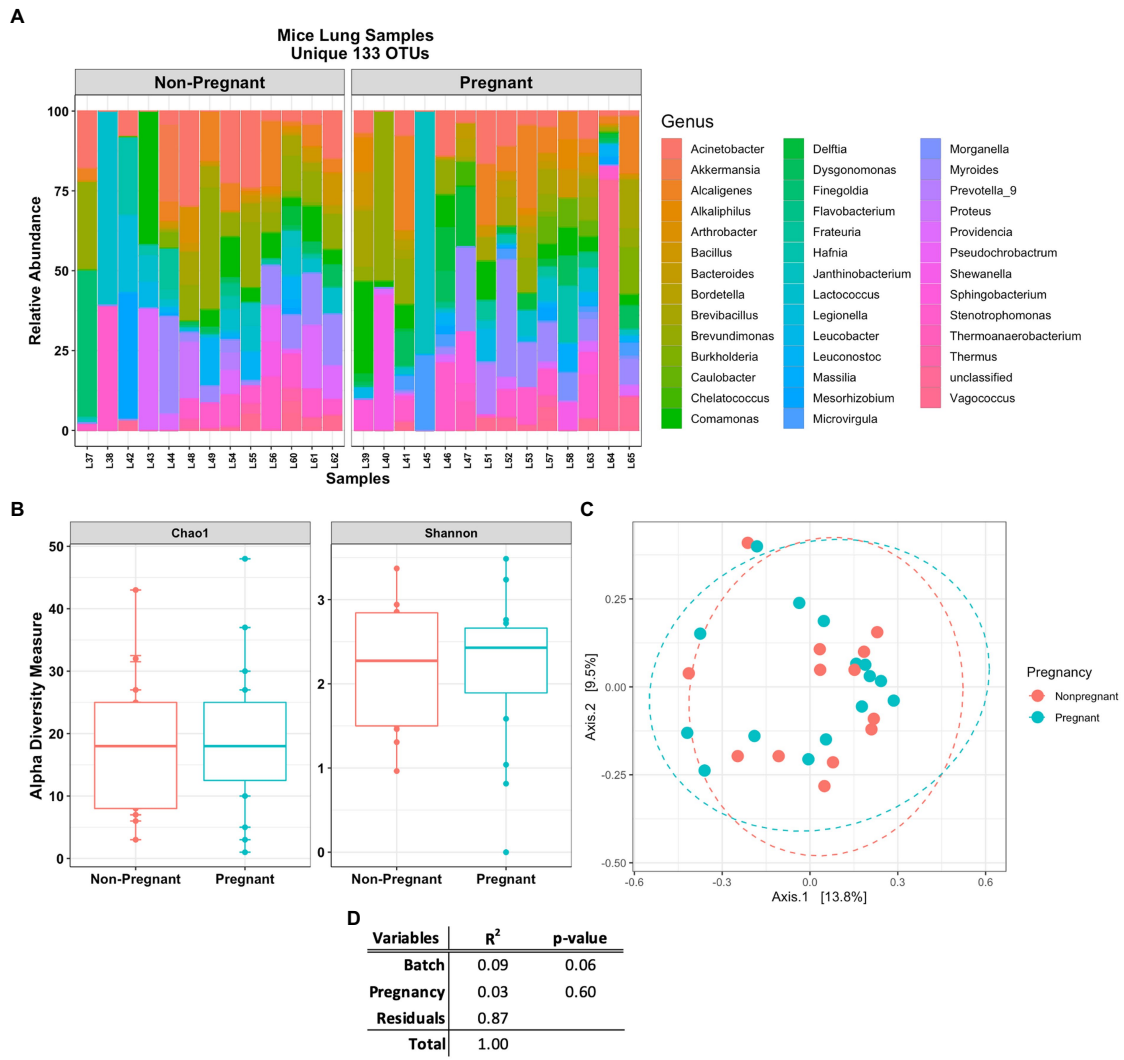
Mice lung samples unique 133 OTUs. **(A)** Abundance plot of pregnant and non-pregnant mice lung samples top 50 unique OTUs. The unique lung microbiome was composed of environmental- and host-associated taxon. **(B)** Comparing alpha and **(C)** beta (unweighted Bray-Curtis PCoA) diversity index of the pregnant and non-pregnant groups. **(D)** Statistical summary of the PERMANOVA test comparing pregnancy and DNA batch extraction variables, no unique microbial signature was observed for the pregnancy variable in the unique lung microbiome.

The Venn diagram in Figure 3C shows that lung and stool data share 413 OTUs. It is estimated that about 49% of the total detected OTUs are shared between the lung and stool samples (Figure 2C). However, 133 unique OTUs were found exclusively in the lung data. Presuming the distinct lung microbial community comprised the 133 OTUs, we used R package phylosmith to identify and extract these OTUs only present in the lung data. After separating the unique OTUs from the lung data, we observed an increase in abundance of environmental-associated bacteria (e.g., *Acinetobacter, Brevundimonas, Comamonas* and *Legionella* sp.) along with some bacteria commonly isolated from diverse host niches (e.g., *Prevotella, Lactococcus* and *Bordetella* sp.; Figure 4A; Wang et al., 2019; Belheouane et al., 2020; Selway et al., 2020). However, none of the unique microbial signatures (OTUs) showed significance in differential abundance between the pregnant and non-pregnant samples (Supplementary Figure S4). Moreover, no significant differences were observed among the two groups in the diversity measures or the permutation test (Figures 4B–D). These results contrast with those obtained prior to removal of suggested stool-associated bacteria were pregnancy led to a significant change in lung samples (Figures 3E, 4D). In summary, in this study pregnancy may only contribute to variance in the overall sequences obtained in the stool and lung microbiome, but not in the unique lung microbiota.

## Discussion

Our results showed that pregnancy influenced the lung and gut microbiome. As established, the murine gut microbiome is largely composed of Bacteroidetes and Firmicutes, while the murine lung microbiome is dominated by Firmicutes, Proteobacteria, and Bacteroidetes; our results are consistent with these findings by others (Yun et al., 2014; Singh et al., 2017; Kostric et al., 2018; Smith, 2019). However, here we report an increase in Firmicutes over Bacteroidetes in the stool microbiome of pregnant mice (Elderman et al., 2018; Faas et al., 2019). Changes to the gut microbiome during pregnancy have been previously reported in humans (Koren et al., 2012; Goltsman et al., 2018). In mice the lung and gut microbiome has been reported (Yun et al., 2014; Singh et al., 2017; Goltsman et al., 2018; Kostric et al., 2018; Wang et al., 2019; Selway et al., 2020), however data on the effects of pregnancy and both sites are still lacking. This report compositionally compares the mice lung and gut microbiomes, describing seeding of the gut microbiota into the lung environment (possibly *via* non-viable bacteria). Furthermore, describing the differential changes in the mice lung microbiome during pregnancy. Nonetheless, seeding of the gut microbiota in the lungs is less likely to occur in human due to hygiene practices and the lack of coprophagia frequently observed in rodents. Of note, seeding of stool-associate bacteria into the lungs could be a common occurrence in animal models. Thus, our study aims to further discuss and interpret the role of the microbiome in pregnant and non-pregnant mouse model and the role of gut bacteria when studying the pulmonary setting.

The overall gut microbial composition of pregnant and non-pregnant mice was distinct. Like other studies (Elderman et al., 2018; Faas et al., 2019), we observed an increase in beneficial bacteria, Lachnospiraceae and Lactobacillaceae, in the pregnant mice stool samples. These potential probiotic bacteria are associated to the production of SCFAs (e.g., acetate, propionate and butyrate), immunomodulatory metabolites that can regulate inflammation from distant mucosal sites (Budden et al., 2017, 2019; Dang and Marsland, 2019). One study showed high-fiber diets during pregnancy elevate serum SCFAs and positively influence metabolic changes such as glucose metabolism, maternal adipokines and airway inflammation (Priyadarshini et al., 2014; Trompette et al., 2014). In our data, functional prediction analysis using R package Tax4fun estimated that SCFAs pathways were more abundant in the pregnant mice stool samples (Supplementary Figure S5). Thus, high abundance of Lachnospiraceae and Lactobacillaceae observed in the pregnant mice gut microbiome could potentially be regulating the airway microbiome and the inflammatory state of this organ (e.g., lung-gut axis). This increase in beneficial (anti-inflammatory) gut bacteria could be a response to the proinflammatory state during the third trimester of pregnancy (Mor et al., 2011). Our data also suggests that pregnancy leads to a reduction in possible pathobionts (e.g., Streptococcus), thus reducing possible pathogenesis associated with colonizing bacteria. To further understand the host–microbe interaction of the gut-lung axis during pregnancy a multi-omic approach (e.g., metagenomics, metatrascriptomics and metabolomics) is required.

Lachnospiraceae and Lactobacillaceae are part of the core gut microbiome that colonizes the intestinal lumen at birth (Vacca et al., 2020). The fact that these potential beneficial gut microbes were detected in mice lungs, supports the concept that seeding of the lung microbiome occurs from the gut microbiota (Bogatyrev et al., 2020). This is suggested by the large detection of shared OTUs between the lung and stool data. Hence, detection of obligate anaerobic microorganisms in an oxygenated environment could be present as inactive (e.g., endospore forming Clostridiales) or dead-cell DNA (Hentges, 1996). Thus, the lung microbiome is influenced by the external environment. Potentially, the gut microbiome could directly (e.g., bacterial seeding) or indirectly (e.g., metabolites) modulate the airway microbiome. Upon closer inspection, the lung microbiome was composed largely of host-associated microbes and not environmental contaminants. Stool-associated microbes (e.g., Lachnospiraceae and Lactobacillaceae) were abundant in the lung microbiome, particularly in pregnant mice. Regarding the limitations of detections using a single hypervariable region 16S rRNA gene, species level of identification is rare (Callahan et al., 2019). However, we were able to identify host specific microbes at a relatively confident percentage of identification using the V4 hypervariable which has an optimal identification threshold at >99% (Edgar, 2018). For instance, *L. gasseri, L. intestinalis,* and *L. mucosae* were identified in our samples with high confidence of identification (Supplementary Table S1). *Lactobacillus* sp. are mostly mutualistic bacteria of the gastrointestinal and the genitourinary tract of humans and animals (Pena et al., 2004; Goldstein et al., 2015; Petrova et al., 2015; Jia et al., 2020). Additionally, intranasally administered *Lactobacillus* sp. has been shown to improve immune responses against pulmonary infections (Hori et al., 2001; Izumo et al., 2010; Fangous et al., 2021). Most Lactobacillaceae are facultative anaerobes (Zheng et al., 2020), lactobacilli can live short-term in the lung environment, observing complete clearance at 7 days post installation (Gabryszewski et al., 2011). Although *Lactobacillus* sp. is regarded as a probiotic gut-associated bacterium, its beneficial properties in the lung environment should be further studied, most importantly during pregnancy.

*Streptococcus* sp. was significantly abundant among the non-pregnant lung samples. Streptococci are host-associated microbes and are a diverse group of bacteria. Depending on the species, streptococci have been isolated from the skin, oral cavity, gastrointestinal and urogenital tract of humans and animals, while some are capable of colonizing and infecting the respiratory tract (e.g., *S. pneumoniae* and *S. pyrogenes*) (Patterson, 1996; Bessen, 2009; Weiser et al., 2018). Of interest, Group B *Streptococcus* (GBS), *Streptococcus agalactiae,* were identified in our samples (Supplementary Table S1). Depending on the strain, GBS could be a common veterinary pathogen or a harmless human commensal (Raabe and Shane, 2019; Gori et al., 2020). However, GBS is also a pathobiont, and it has been associated to cases of invasive infections in newborns, pregnant women, and immunocompromised individuals (Shabayek and Spellerberg, 2018; Freudenhammer et al., 2021; Mejia et al., 2022). Of interest, different *Lactobacillus* sp. have shown to inhibit the growth of *Streptococcus* sp. (Wasfi et al., 2018; Lee et al., 2021; Kim et al., 2022). *Lactobacillus* sp. were dominant in the pregnant mice and *Streptococcus* sp. were dominant in the non-pregnant mice lungs, suggesting that the increase in *Lactobacillus* sp. may be a mechanism to prevent infection during pregnancy (Kostric et al., 2018).

The lung microbiome showed evidence of seeding from stool microbes. According to Cardona et al. (2018), while most exogenous bacteria influence the host-microbiota, they do not result in colonization of the host niches (Cardona et al., 2018). Exogenous bacteria, either from an environmental source or from different host sites, are mostly transient in the respiratory tract (Charlson et al., 2011; Dickson et al., 2015; Cardona et al., 2018). In human studies, the lung microbiome is an open system continuously influenced by external environmental factors (Bogaert et al., 2011; Dickson et al., 2015; Birzele et al., 2017). The lung microbiome of healthy individuals is dynamic, it is subjected to constant microbial migration (*via* microaspirations or mucociliary transport) and elimination (e.g., alveolar macrophages), thus reducing the likelihood of exogenous bacteria to reside long-term in the lung environment (Dickson et al., 2015; Huffnagle et al., 2017). The same can be said for murine lung microbiome (Yun et al., 2014; Singh et al., 2017; Kostric et al., 2018; Smith, 2019), as the lung microbiome can be influenced by the surrounding environment (e.g., housing conditions) and mice habits (e.g., coprophagia). In conclusion, pregnancy changes in the mouse lung and gut microbiomes.

## Data availability statement

The datasets presented in this study can be found in online repositories. The names of the repository/repositories and accession number(s) can be found at: https://www.ncbi.nlm.nih.gov/, PRJNA837989.

## Ethics statement

The animal study was reviewed and approved by Institutional Animal Care and Use Committee approved by the Rhode Island Hospital.

## Author contributions

RW-R, AT, MR, and CK carried out the experiments. RW-R, HS, LO, AF, and NG-J contributed to the design and conceptualization of the project. RW-R, HS, AF, and NG-J wrote and edited the manuscript. All authors contributed to the article and approved the submitted version.

## Funding

This study was supported by the National Institutes for Health (NIH) awards AI148722-01A1 and ES030227.

## Conflict of interest

The authors declare that the research was conducted in the absence of any commercial or financial relationships that could be construed as a potential conflict of interest.

## Publisher’s note

All claims expressed in this article are solely those of the authors and do not necessarily represent those of their affiliated organizations, or those of the publisher, the editors and the reviewers. Any product that may be evaluated in this article, or claim that may be made by its manufacturer, is not guaranteed or endorsed by the publisher.
