# Supplementary material for: Gut and lung microbiome profiles in pregnant mice
Source: Front Microbiol. 2022 Dec 12;13:946779. doi: 10.3389/fmicb.2022.946779 (PMC9791091; doi:10.3389/fmicb.2022.946779)
Supplement: Supplementary file 1 [file Data_Sheet_1.DOCX]

**Supplemental Data**:


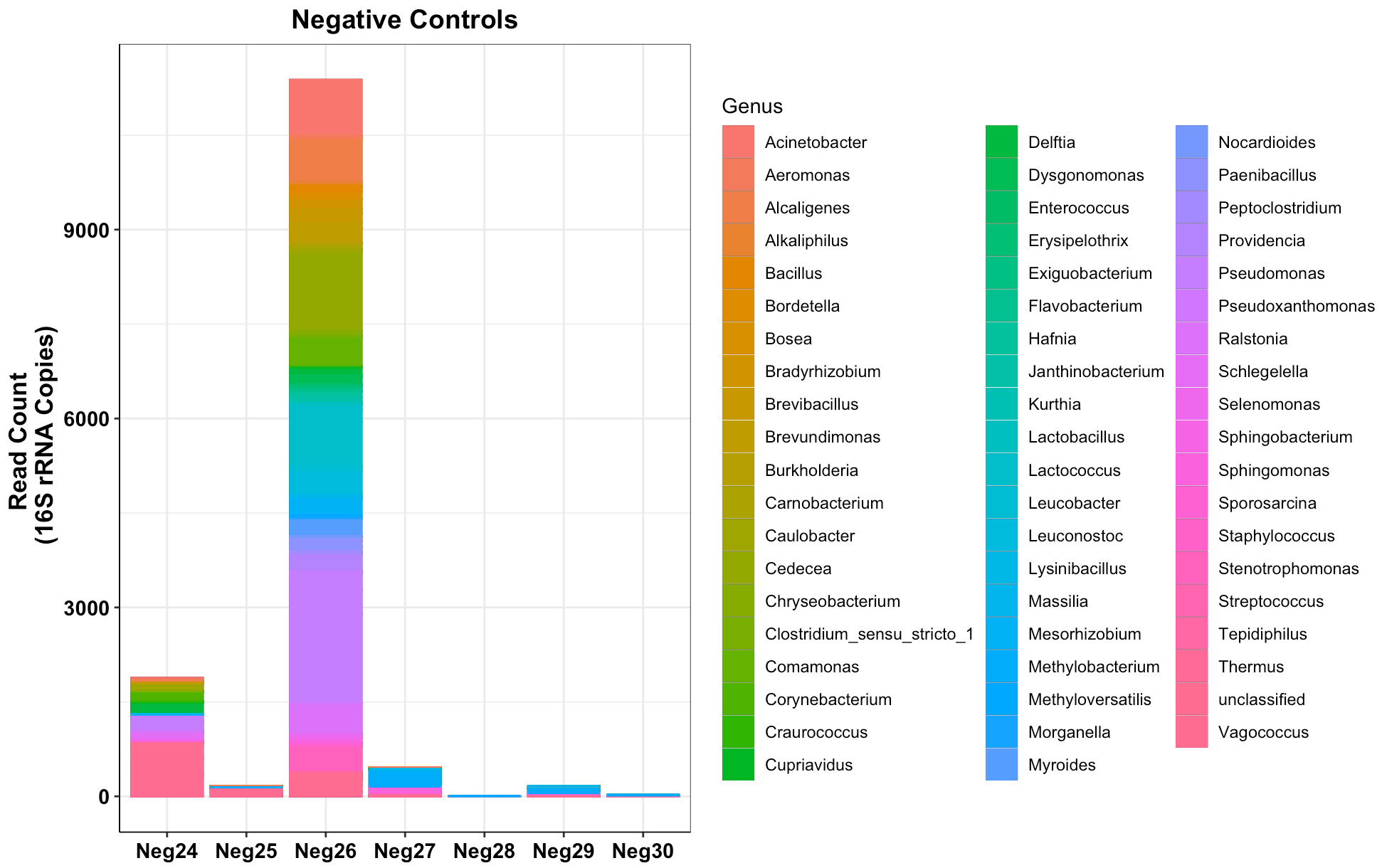


**Fig. S1 Negative controls total read count (16S rRNA Copies) after quality control and filtering through the 16S pipeline.** Overall, negative controls show trace amount of contamination from environmental, or water contaminates. Example, *Sphingomonas*, *Pseudomonas* and *Ralstonia* sp. are commonly isolated form water or soil sources. Negative control#26 showed slight contamination from lung samples during batch extractions.


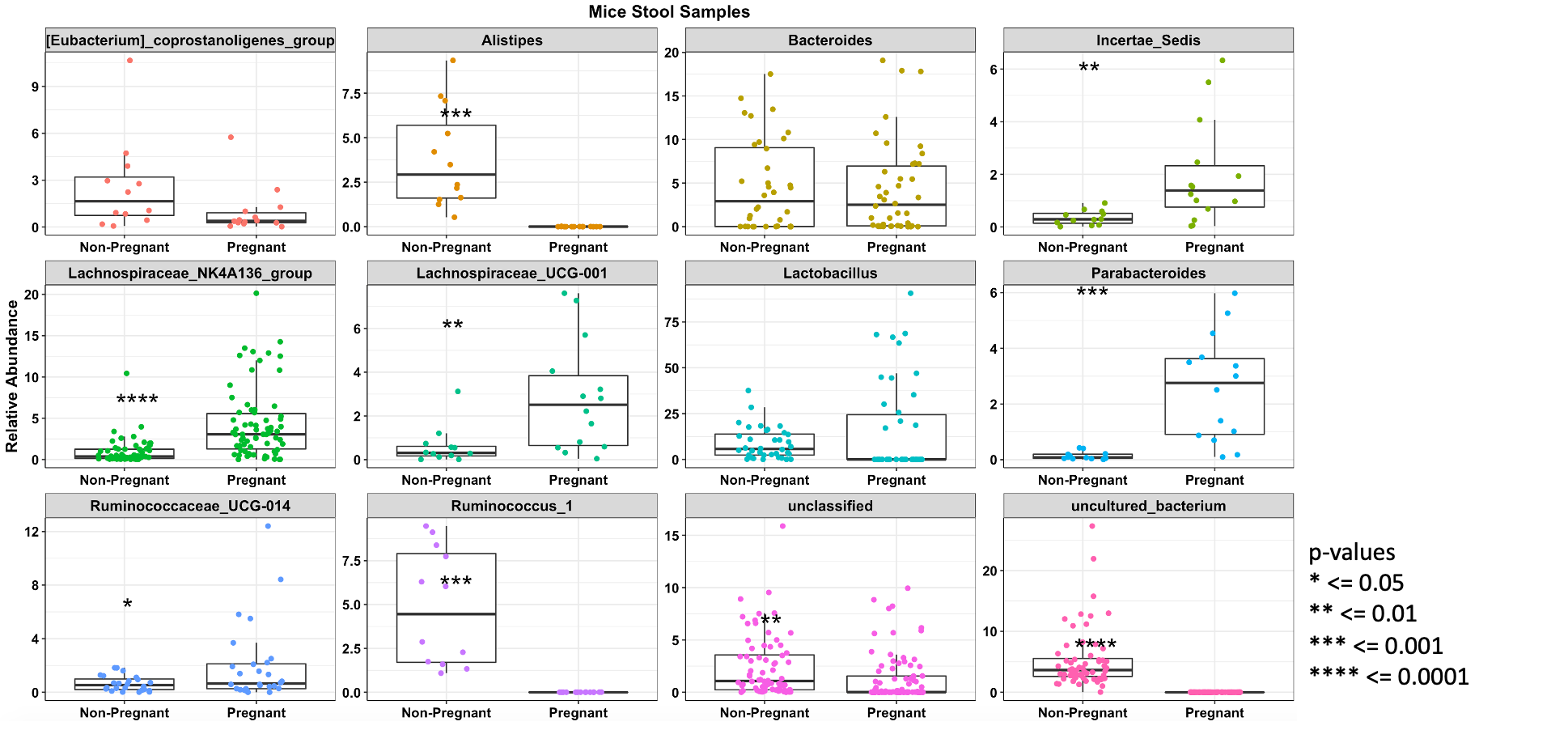


**Fig S2. Comparing microbial composition at Genus level of pregnant and non-pregnant mice stool samples.** The plot present the top 30 OTUs identified in the sample data. T-test was performed to determine the differential significance between pregnant and non-pregnant samples. We observed similar results in the differential abundance detected by LeFSe (see Fig S7). Lachnospiraceae (NK4A136 and UCG-001) was significantly higher in pregnant mice whereas *Alistipes* and *Ruminococcus* sp. was significantly higher in non-pregnant mice stool samples. Noticeably, non-pregnant mice had a higher abundance of unclassified and uncultured bacteria (taxonomic limitations of 16S).


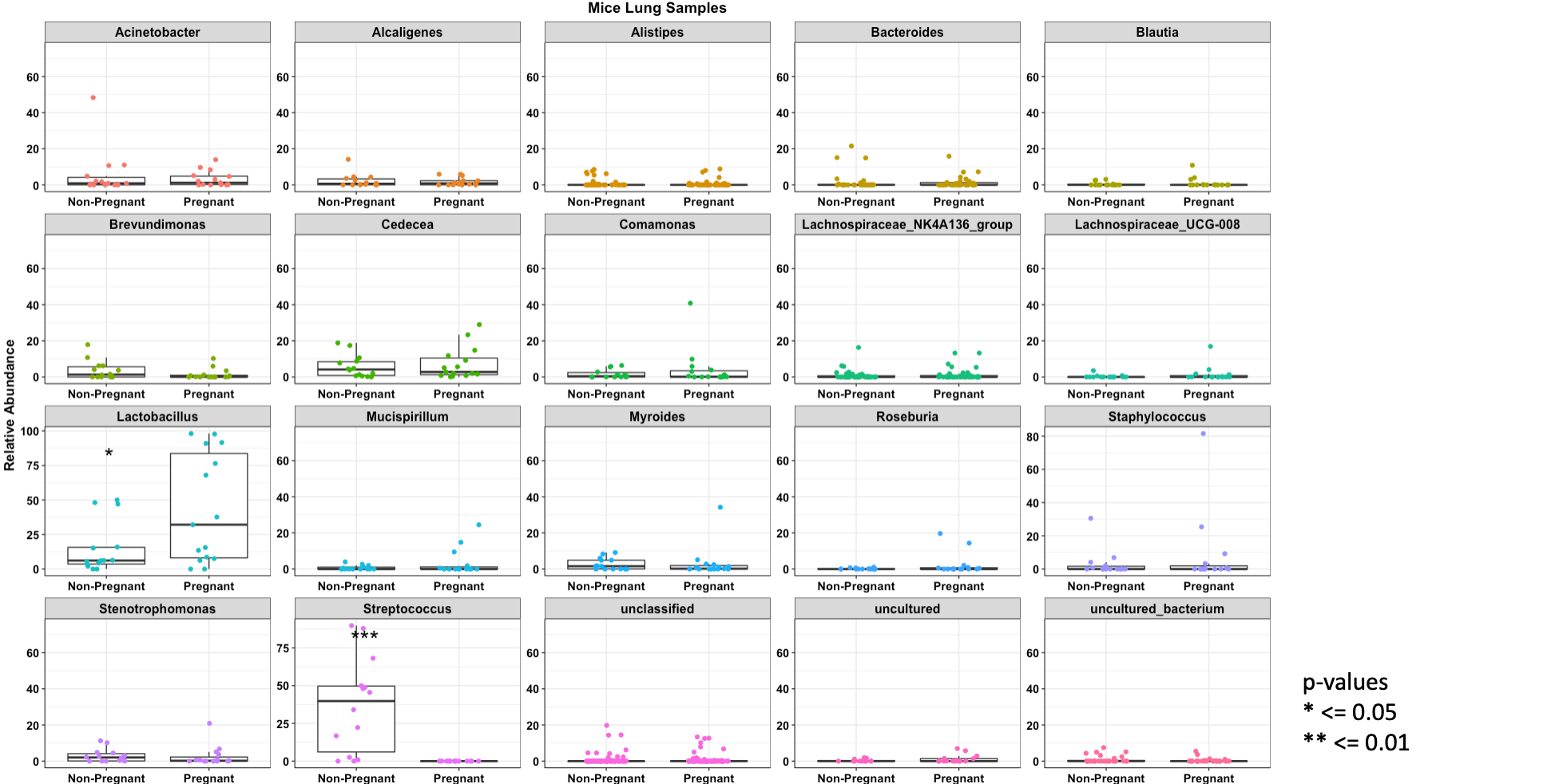


**Fig S3. Comparing microbial composition at Genus level of pregnant and non-pregnant mice lung samples.** The plot presents the top 30 OTUs identified in the sample data. T-test was performed to determine the differential significance between pregnant and non-pregnant samples. *Lactobacillus* sp. was significantly higher in pregnant mice whereas *Streptococcus* sp. was significantly higher in non-pregnant mice lung samples.


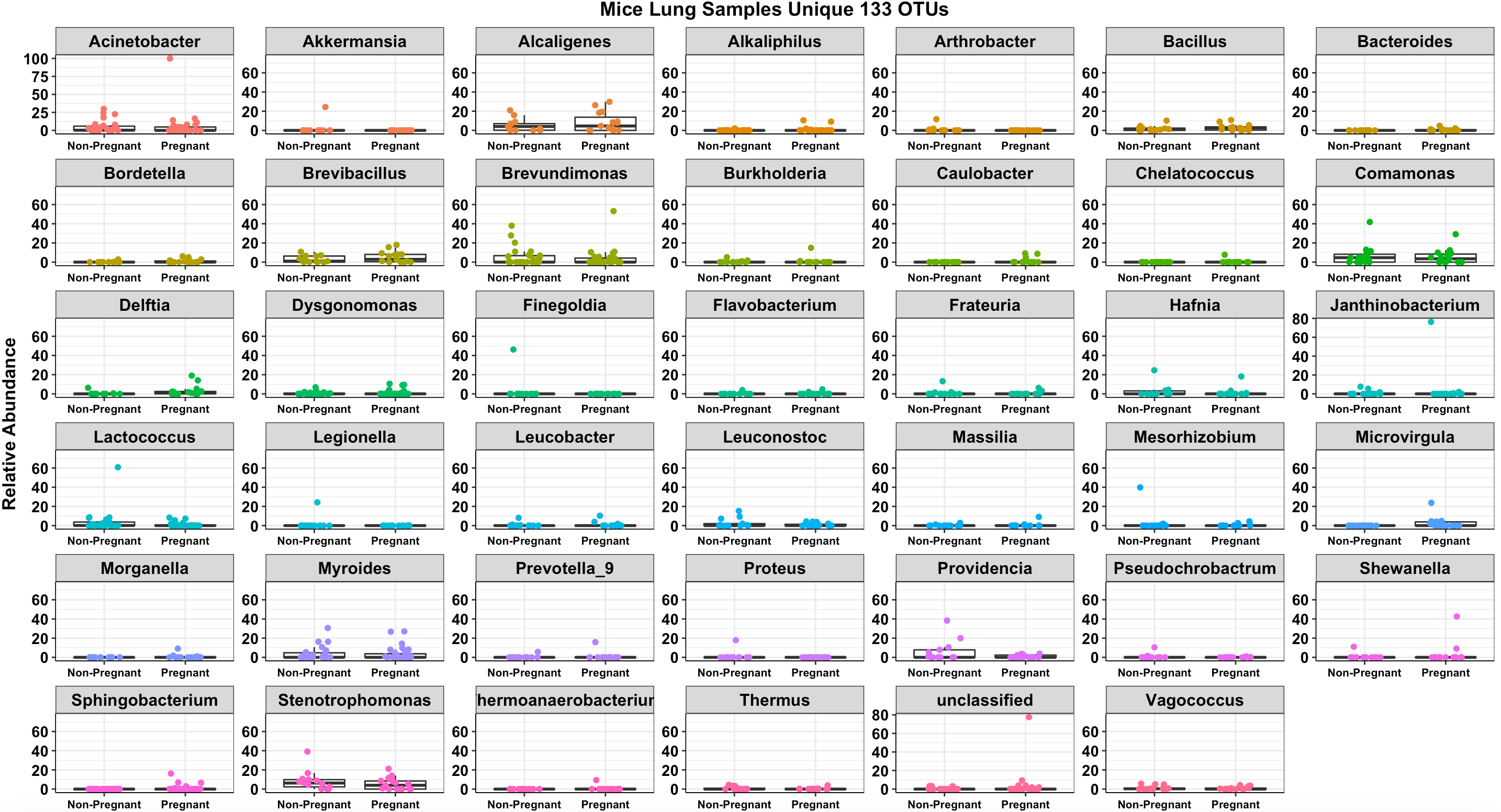


**Fig S4. Comparing microbial composition at Genus level of pregnant and non-pregnant mice lung samples top 133 unique OTUs.** After removing stool microbiota contamination, no unique microbial signatures were observed between the pregnant and non-pregnant samples.


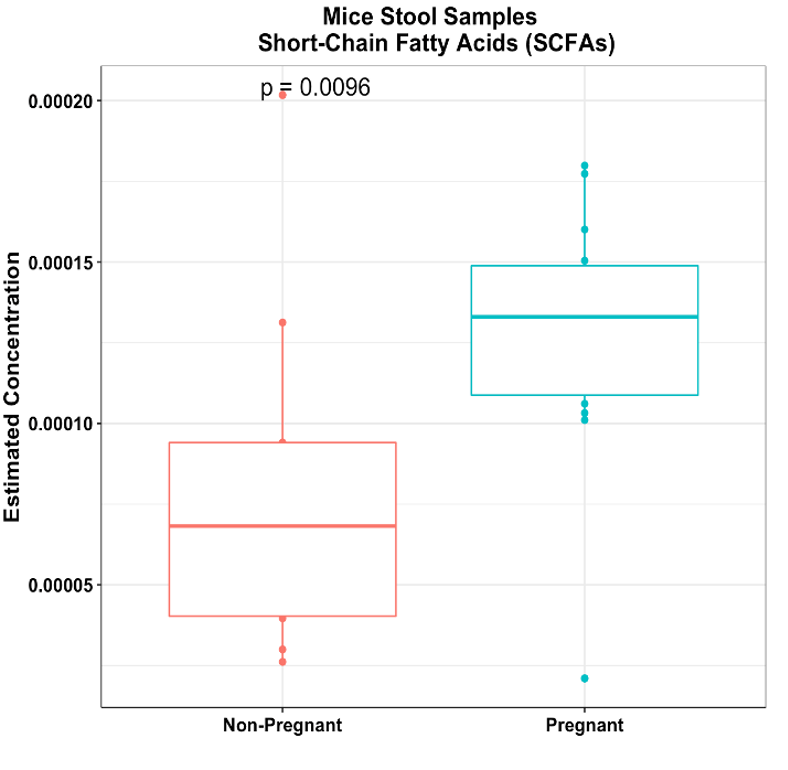


**Fig S5. Comparing functional prediction of pregnant and non-pregnant mice lung and stool sample using R package Tax4Fun.** Estimates that **A)** short-chain fatty acids have a higher concentration in pregnant mice samples.


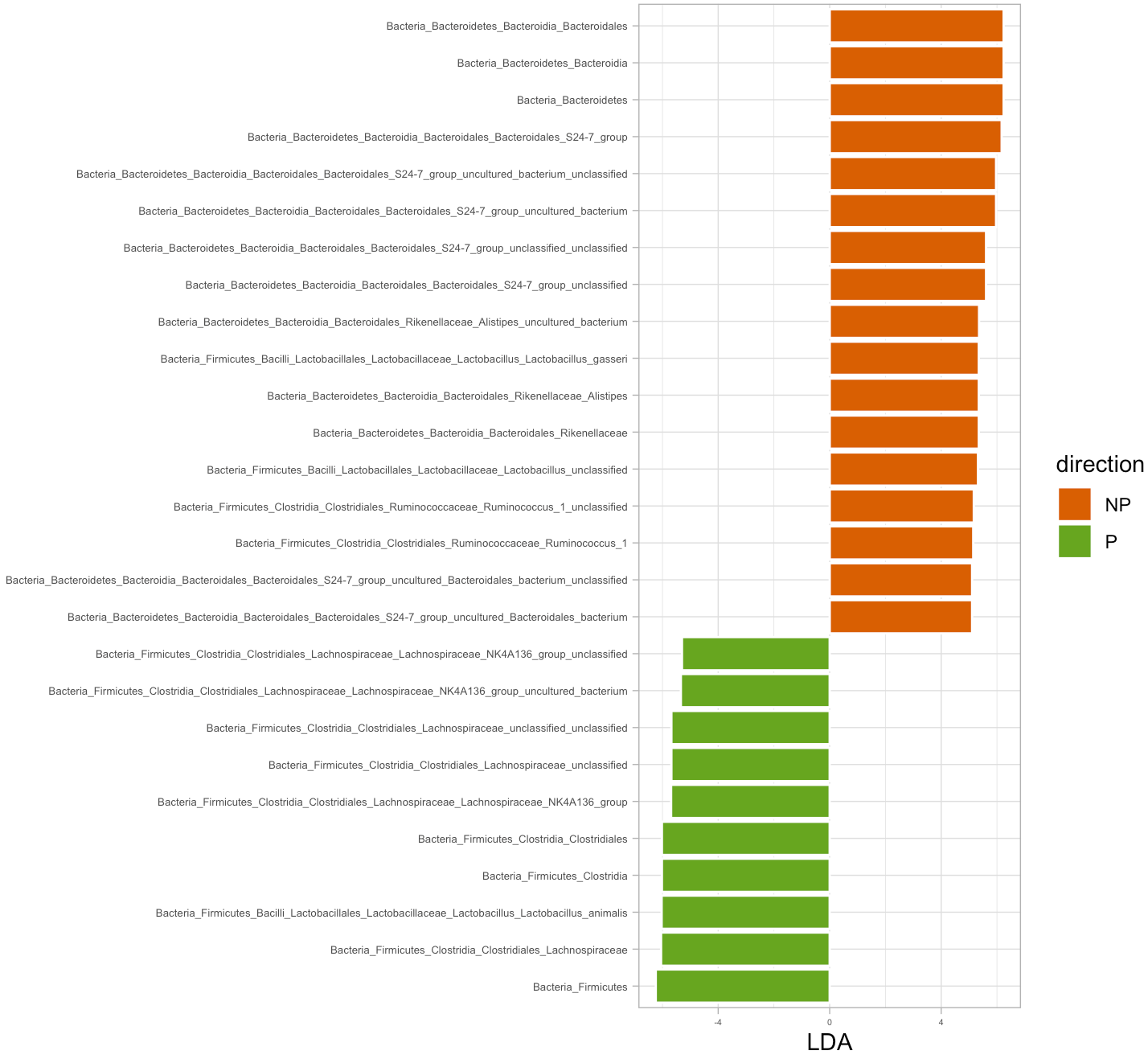


**Fig S7. Differential abundance plot of pregnant and non-pregnant mice stool samples.** The plot is a LDA (Linear Discriminant Analysis) generated by LeFSe which determines effect size of the LDA, cutoff for p-value and FDR set to <0.05.
